# Umfassender Literaturüberblick über die Anwendung der otologisch-chirurgischen Planungssoftware OTOPLAN® bei der Cochleaimplantation

**DOI:** 10.1007/s00106-024-01461-8

**Published:** 2024-04-08

**Authors:** Franz-Tassilo Müller-Graff, Björn Spahn, David P. Herrmann, Anja Kurz, Johannes Voelker, Rudolf Hagen, Kristen Rak

**Affiliations:** Klinik und Poliklinik für Hals‑, Nasen- und Ohrenkrankheiten, plastische und ästhetische Operationen, Universitätsklinikum Würzburg, Josef-Schneider-Straße 11, 97080 Würzburg, Deutschland

**Keywords:** Anatomiebasiertes Fitting, Computertomographie-basierte Software Ohr/Cochlea, Bildgebungsmodalitäten (MRT, Computertomographie [flat-panel volume CT]), Cochleäre Länge, Computersimulation, Anatomy based fitting, Computed tomography-based software cochlear, Imaging modalities (MRI, computer tomography [flat-panel volume CT]), Cochlear duct length, Computer simulation

## Abstract

**Hintergrund:**

Die Größe der menschlichen Cochlea, gemessen am Durchmesser der Basalwindung, schwankt zwischen 7 und 11 mm. Im Rahmen einer Hörrehabilitation durch ein Cochleaimplantat ist diese für die individuelle Zuordnung der Frequenzbänder und die Wahl der Elektrodenlänge von Bedeutung. OTOPLAN® (CAScination AG [Bern, Schweiz] in Kooperation mit MED-EL [Innsbruck, Österreich]) ist ein Softwaretool mit CE-Kennzeichnung für klinische Anwendungen in der Cochleaimplantat(CI)-Behandlung, welches die Vorplanung auf Grundlage der cochleären Größenparameter durchführt. Ziel dieser Literaturübersicht ist es, alle veröffentlichten Studien über die Anwendung von OTOPLAN® zu erfassen.

**Materialien und Methoden:**

Die PRISMA-Richtlinien (Preferred Reporting Items for Systematic Reviews and Meta-Analyses) wurden angewandt, um relevante Studien zu identifizieren, die zwischen Januar 2015 und Februar 2023 in der Suchmaschine PubMed veröffentlicht wurden (unter Verwendung der Suchbegriffe „otoplan“ [Titel/Abstract] OR „anatomy-based fitting“ [Titel/Abstract] OR „otological software tool“ [Titel/Abstract] OR „computed tomography-based software AND cochlear“ [Titel/Abstract]).

**Ergebnisse:**

Bei der systematischen Durchsicht der Literatur wurden 32 Studien über den klinischen Einsatz von OTOPLAN® bei der CI-Behandlung gefunden. Die meisten Studien wurden von deutschen Arbeitsgruppen publiziert (7 von 32), gefolgt von Italien (5), Saudi-Arabien (4), USA (4) und Belgien (3). So stammten je 2 Studien aus Österreich und China, gefolgt von jeweils 1 Studie aus Frankreich, Indien, Norwegen, Südkorea und der Schweiz. In den meisten Studien (22) wurde OTOPLAN® zur Beurteilung der Cochleagröße verwendet, gefolgt von der Visualisierung der Elektrodenposition anhand postoperativer Bilder (5), der dreidimensionalen (3-D-)Segmentierung der Felsenbeinstrukturen (4), der Planung der Elektrodeneinführungstrajektorie (3), der Erstellung einer patientenspezifischen Frequenzbandzuordnung (3), der Planung eines sicheren Bohrpfads durch den Recessus facialis (3), und der Messung von Felsenbeinstrukturen (1).

**Schlussfolgerung:**

OTOPLAN® ist bisher der einzige DICOM-Viewer mit CE-Kennzeichnung im CI-Bereich, der prä-, intra- und postoperative Bilder mit den genannten Anwendungen verarbeiten kann.

## Abstract (en)

**Background:**

The size of the human cochlear, measured by the diameter of the basal turn, varies between 7 and 11 mm. For hearing rehabilitation with cochlear implants (CI), the size of the cochlear influences the individual frequency map and the choice of electrode length. OTOPLAN® (CAScination AG [Bern, Switzerland] in cooperation with MED-EL [Innsbruck, Austria]) is a software tool with CE marking for clinical applications in CI treatment which allows for precise pre-planning based on cochlear size. This literature review aims to analyze all published data on the application of OTOPLAN®.

**Materials and methods:**

The Preferred Reporting Items for Systematic Reviews and Meta-Analyses (PRISMA) guidelines were applied to identify relevant studies published in the PubMed search engine between January 2015 and February 2023 using the search terms “otoplan” [title/abstract] OR “anatomy-based fitting” [title/abstract] OR “otological software tool” [title/abstract] OR “computed tomography-based software AND cochlear” [title/abstract].

**Results:**

The systematic review of the literature identified 32 studies on clinical use of OTOPLAN® in CI treatment. Most studies were reported from Germany (7 out of 32), followed by Italy (5), Saudi Arabia (4), the USA (4), and Belgium (3); 2 studies each were from Austria and China, and 1 study from France, India, Norway, South Korea, and Switzerland. In the majority of studies (22), OTOPLAN® was used to assess cochlear size, followed by visualizing the electrode position using postoperative images (5), three-dimensional segmentation of temporal bone structures (4), planning the electrode insertion trajectory (3), creating a patient-specific frequency map (3), planning of a safe drilling path through the facial recess (3), and measuring of temporal bone structures (1).

**Conclusion:**

To date, OTOPLAN® is the only DICOM viewer with CE marking in the CI field that can process pre-, intra-, and postoperative images in the abovementioned applications.

## Aufbau eines Cochleaimplantats

Die Cochleaimplantation ist eine bewährte Technologie, die seit mehr als 40 Jahren zur Wiederherstellung des Hörvermögens bei sensorineuralem Hörverlust eingesetzt wird [1]. Bis heute (zum Zeitpunkt der Ausarbeitung dieses Artikels) wurden insgesamt 900.000 Cochleaimplantate (CI) erfolgreich implantiert [54].

Ein CI besteht aus Audioprozessor, implantierbarem elektronischem Schaltkreis sowie intracochleärer Elektrode

Ein CI besteht aus einem extern getragenen Audioprozessor und einem implantierbaren elektronischen Schaltkreis, der von einem Titangehäuse umschlossen ist, sowie einer intracochleären Elektrode. Im Audioprozessor wird das über das integrierte Mikrofon aufgenommene Schallsignal in frequenzspezifische digitale Signale umgewandelt, die über eine induktive Verbindung an die implantierbare Elektronik weitergeleitet werden. Die implantierte Elektronik wandelt diese frequenzspezifischen digitalen Signale in frequenzangepasste elektrische Impulse um, die dann über ein intracochleäres Elektrodenarray, das längs in der Scala tympani (ST) platziert wird, an die nervalen Strukturen in der Cochlea abgegeben werden. Diese neuralen Elemente, die in der Cochlea tonotopisch angeordnet sind, mit höheren Frequenzen am basalen Ende, niedrigeren Frequenzen am apikalen Ende und dazwischen liegenden mittleren Frequenzen, werden durch das elektrische Signal depolarisiert und übertragen die entsprechende Information an den Hörnerv, der es an den Hörkortex weiterleitet, wo es als Klang wahrgenommen wird [14].

## Voraussetzungen für den Operationserfolg

Die chirurgische Platzierung der CI-Elektrode innerhalb der Cochlea zur Schaffung einer effektiven Elektroden-Nerven-Schnittstelle ist einer der Schlüsselfaktoren für eine erfolgreiche CI-Behandlung [15]. Die individuelle cochleäre Größe ist der Grund dafür, dass eine Elektrode unterschiedlich tief in der Cochlea zu liegen kommt [21]. Es wurde berichtet, dass eine gute Übereinstimmung in der Länge zwischen Elektrode und ST zu einer guten Übereinstimmung in der Tonhöhenwahrnehmung zwischen der natürlich hörenden Seite und der CI-versorgten Seite bei einseitig gehörlosen Personen führt [50]. Einschränkend muss angemerkt werden, dass diese Daten nur mit Elektroden eines CI-Herstellers (Fa. MED-EL, Innsbruck, Österreich) und in einer kleinen Stichprobengröße durchgeführt wurden. Eine längere Elektrode, die den größten Teil der Cochlea abdeckt, führt auch bei hochgradig schwerhörigen Personen zu besseren Hörergebnissen als eine kurze Elektrode, die nur die basale Windung der Cochlea abdeckt [8, 9, 20, 25, 45]. Dies kann bei jedem CI-Kandidaten sicher und konsistent erreicht werden, wenn die cochleäre Länge präoperativ bekannt ist, was dem Chirurgen hilft, eine Elektrode mit der geeigneten Länge auszuwählen.

Über anatomische Variationen in Größe und Form der menschlichen Cochlea wurde in der Literatur bereits ausführlich publiziert. Im Jahr 2005 berichtete der französische Radiologe Dr. Bernard Escude, dass der grundlegende Cochleaparameter, der Basalwindungsdurchmesser (A-Wert) in der sog. Cochleaansicht („cochlear view“, d. h. in der koronalen Schrägansicht), die cochleäre Lange („cochlear duct length“, CDL) entlang der äußeren Seitenwand („lateral wall“, LW) vom Eingang des runden Fensters („round window“, RW) bis zu einer beliebigen Einschubtiefe (CDL_LW_) vorhersagen kann [19]. Einschränkend ist aber auf eine erhebliche Interratervariabilität bei dieser Formel hinzuweisen [7]. Im weiteren Verlauf gab es weitere Berichte über fein abgestimmte mathematische Gleichungen zur Vorhersage der CDL entlang der Basilarmembran (BM; CDL_BM_) oder des Corti-Organs (OC; CDL_OC_). Dies ist relevanter, da die geraden, nicht vorgeformten Elektroden der lateralen Wand (engl. „straight lateral wall electrodes“) i. d. R. direkt unter der BM oder dem OC sitzen [32, 52]. Die Greenwood-Frequenzfunktion berücksichtigt auch die CDL entlang des OC, um die patientenspezifische Frequenzbandzuordnung zu erhalten [56].

Eine genaue Messung der Cochleagröße hilft, (i) die CDL abzuschätzen, (ii) eine patientenspezifische Frequenzbandzuordnung zu erstellen, (iii) diejenige Insertionstiefe zu bestimmen, bei der das Restgehör am apikalen Ende der Cochlea beginnt, (iv) eine Elektrodenlänge an die CDL anzupassen und (v) das Restgehör zu bestimmen. Die Genauigkeit und Reproduzierbarkeit der Größenmessung der Cochlea durch mehrere Beobachter spielt eine entscheidende Rolle für den Gesamterfolg der cochleären Größenmessung in der klinischen Forschung.

Genauigkeit und Reproduzierbarkeit sind entscheidend für den Gesamterfolg cochleärer Größenmessung

Mit der Markteinführung von OTOPLAN® (CAScination AG [Bern, Schweiz] in Kooperation mit MED-EL [Innsbruck, Österreich]) im Jahr 2018 und der CE-Kennzeichnung wurde ein spezielles Software-Tool für die CI-Vorplanung eingeführt, das (i) die Messung der Cochleagröße vereinfacht, (ii) die Visualisierung patientenspezifischer Frequenzbandzuordnungen ermöglicht, (iii) die am besten passende Elektrodenlänge simuliert und (iv) die Position einer eingeführten Elektrode bei der Auswertung der postoperativen Bildgebung kontrolliert. Die eigenen Erfahrungen der Autoren mit der klinischen Anwendung der Software OTOPLAN® haben sie dazu veranlasst, in der Literatur zu recherchieren, wie effektiv OTOPLAN® bisher klinisch eingesetzt wurde.

## Methoden

Ziel der vorliegenden Übersichtsarbeit war es, die klinischen Anwendungen von OTOPLAN® im CI-Bereich zu ermitteln.

### Suchstrategie

Die Überprüfung wurde gemäß den PRISMA-Richtlinien (Preferred Reporting Items for Systematic Reviews and Meta-Analyses) [40] durchgeführt, wobei PubMed als Suchmaschine verwendet wurde. Von Anfang Januar 2015 bis Ende Februar 2023 publizierte Artikel wurden in die Suche einbezogen. Dieser Zeitraum markiert die Zeit nach der Einführung von OTOPLAN® als digitales Hilfsmittel im Jahr 2015.

### Studienauswahl

Die relevanten Publikationen wurden von einem Autor anhand vordefinierter Suchbegriffe extrahiert. Es wurden breite Suchkriterien verwendet, um möglichst viele veröffentlichte Artikel zu erfassen. Die Suchbegriffe waren: („otoplan“ [Titel/Abstract] OR „anatomy-based fitting“ [Titel/Abstract] OR „otological software tool“ [Titel/Abstract] OR „computed tomography-based software AND cochlear“ [Titel/Abstract]). Übersichtsartikel, die den Begriff OTOPLAN® in der Zusammenfassung enthielten, wurden von dieser systematischen Literaturübersicht ausgeschlossen.

Die Titel und/oder Zusammenfassungen wurden manuell gesichtet, um Studien zu identifizieren, welche die Ein- und Ausschlusskriterien erfüllten. Dabei überprüften 2 Autoren (FTMG und KR) die Artikel unabhängig voneinander. Die aus den relevanten Artikeln extrahierten Informationen wurden verwendet, um eine vordefinierte Excel-Tabelle zu füllen. Die Tabelle enthielt die PubMed-ID, die Autoren des Artikels, das Jahr der Veröffentlichung, das Herkunftsland, die Art der Studie, das Ziel der Studie mit OTOPLAN®, die Anzahl der Studienteilnehmer, die analysierte Anatomie des Felsenbeins und das Alter der CI-Patienten. Unstimmigkeiten zwischen den Gutachtern über die gesammelten Daten wurden durch gemeinsame Diskussion und Konsens gelöst. Diese betrafen insbesondere die Zuordnung einzelner Studien zu den verschiedenen Anwendungsgebieten der Software, da manche Studien sich mit mehreren Funktionen gleichzeitig beschäftigten.

## Ergebnisse

Die Suche wurde am 20. Februar 2023 durchgeführt, um alle Studien einzuschließen, die OTOPLAN® zum Zeitpunkt der Ausarbeitung dieses Artikels verwendeten. Alle identifizierten Studien berichteten über eine erfolgreiche Anwendung von OTOPLAN®.

### Beschreibung der Studien

**Abb. 1:**
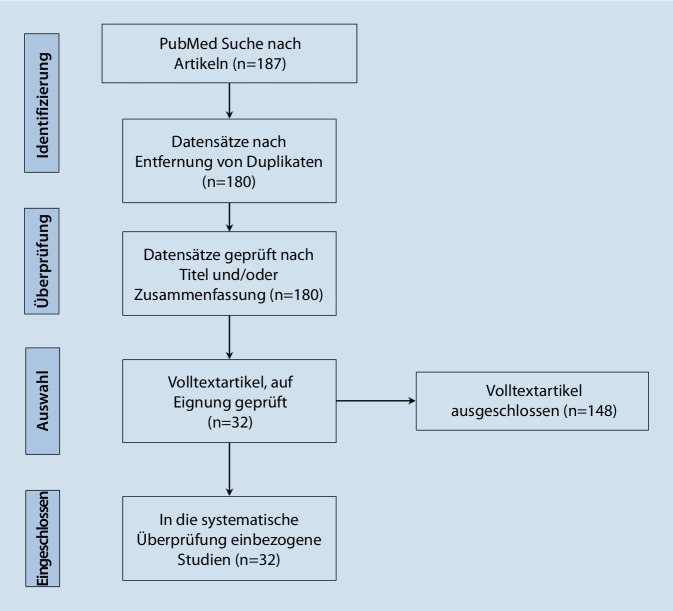
Flussdiagramm zum Prozess der Literaturrecherche anhand der Leitlinien der Preferred Reporting Items for Systematic Reviews and Meta-Analyses (PRISMA)

Die Ein- und Ausschlusskriterien wurden von 187 relevanten Studien zunächst erfüllt. Die Abb. 1 zeigt ein Flussdiagramm, in dem die Anzahl der in jedem Schritt gemäß den PRISMA-Leitlinien identifizierten Studien aufgeführt ist. Nach dem Entfernen von Duplikaten wurden von den verbliebenen 180 Studien nach dem Screening des Titels und/oder der Zusammenfassung insgesamt 148 Studien ausgeschlossen.

Es verblieben insgesamt 32 Studien in der endgültigen systematischen Übersicht

Somit verblieben insgesamt 32 Studien in der endgültigen systematischen Übersicht.

### Demografie

In Tab. 1 sind die demografischen Daten der Studien aufgeführt, die aus den 32 relevanten Veröffentlichungen gesammelt wurden. Bei 23 Publikationen handelte es sich um retrospektive Studien, bei 2 um Kadaverstudien, bei 2 um Fallberichte, bei 2 um prospektive Studien, bei 1 um eine klinische Erprobung, und bei den übrigen wurde die Art der Studie nicht angegeben. Studien wurden von multiplen geografischen Standorten aus verschiedenen Kontinenten publiziert. Ein Maximum von 7 Studien stammte aus Deutschland, 5 aus Italien, je 4 aus Saudi-Arabien und den Vereinigten Staaten, 3 aus Belgien, je 2 aus Österreich und China und je 1 aus Frankreich, Indien, Norwegen, Südkorea und der Schweiz.

**Tab. 1:** Erhebung der demografischen Daten aus den 32 identifizierten Studien

PMID	Autor	Herkunftsland	Studiendesign	Ziel mit OTOPLAN®	Anzahl Probanden	Analysierte Anatomie	Alter der Probanden
26736914	Lu et al. 2015 [47]	Schweiz	Kadaverstudie	Segmentierung des Gesichtsnervs anhand klinischer CT-Bilder	5	Normale Anatomie	Kadaverköpfe
30531645	Lovato et al. 2019 [34]	Italien	Fallbericht	Präoperative Operationsplanung bei einer cochleären Verknöcherung nach einer Meningitis	1	Verknöcherte Cochlea	46 Jahre
32569151	Lovato et al. 2020 [35]	Italien	Prospektiv	Chirurgische Planung der CI-Versorgung bei Patienten mit fortgeschrittener Otosklerose	5	Fortgeschrittene Otosklerose	59,6 Jahre
32209514	Topsakal et al. 2020 [59]	Belgien	Retrospektiv	Vergleich der Trajektorie der Elektrodeneinführung bei verschiedenen chirurgischen Techniken	Keine Angabe	Normale Anatomie	Keine Angabe
32493102	Khurayzi et al. 2020 [28]	Saudi-Arabien	Retrospektiv	Vergleich der A‑Wert-Messung zwischen OTOPLAN® und Standard-DICOM-Viewer	88	Normale Anatomie	1–7 Jahre
32080026	Almuhawas et al. 2020 [3]	Saudi-Arabien	Retrospektiv	Messung von Mastoiddicke und Schädelbreite	92	Normale und malformierte Anatomie	0,5–79 Jahre
34820415	Jablonski et al. 2021 [27]	Norwegen	Kadaverstudie	Zugang zum RW ausschließlich mit dem bildgesteuerten Robotersystem anstelle des manuellen Bohrens zum RW	16	Normale Anatomie	Kadaverköpfe
33273309	Mlynski et al. 2021 [39]	Deutschland	Retrospektiv	Messung der CDL und Korrelation zum postoperativen Sprachverständnis sowie zu ECAP	53	Normale Anatomie	63,6 Jahre
34590531	Cooperman et al. 2021 [13]	USA	Retrospektiv	Schätzung der CDL durch Messung des A‑Werts der Cochlea	61	Normale Anatomie	Erwachsene Patienten
34050805	Spiegel et al. 2021 [55]	Deutschland	Retrospektiv	Schätzung der CDL	180	Normale Anatomie	6,5–90,3 Jahre
33710146	Chen et al. 2021 [11]	China	Retrospektiv	Schätzung der CDL und Vergleich mit der MPR	68	Normale Anatomie	0,6–63,3 Jahre
33492059	Cooperman et al. 2021 [12]	USA	Retrospektiv	Messung der CDL	166	Normale Anatomie	65,63 Jahre
33455125	Niu et al. 2021 [43]	China	Prospektiv	Schätzung der CDL und Wahl der Elektrodenlänge	26	Normale Anatomie	19–71 Jahre
33143454	Andersen et al. 2021 [4]	USA	Retrospektiv	Segmentierung von Mittelohr- und Innenohrstrukturen	9	Normale Anatomie	3–12 Jahre
32826506	Lee et al. 2021 [31]	Südkorea	Retrospektiv	Messung der Cochleaparameter	51	Normale Anatomie	26–112 Monate 12–468 Monate 7–91 Monate
34660683	Auinger et al. 2021 [5]	Österreich	Retrospektiv	Planung des Bohrpfads von der Schädeloberfläche zum Cochleaeingang bei sicherer Durchquerung des Recessus facialis	50	Normale Anatomie	51 ± 23 Jahre
36351223	Kurz et al. 2022 [29]	Deutschland	Retrospektiv	Anwendung der anatomiebasierten Anpassung bei erfahrenen CI-Trägern	3	Normale Anatomie	57, 57, 38 Jahre
36544941	Dhanasingh et al. 2022 [16]	Österreich	Keine Angabe	Die systematische Visualisierung des Innenohrs sowohl in der Cochleaansicht (schräge koronale Ebene) als auch im mittleren modiolären Schnitt (axiale Ebene) in 3 aufeinanderfolgenden Schritten vereinfacht die Identifizierung der Arten von Innenohrfehlbildungen	112	Normale und malformierte Anatomie	Keine Angabe
34101009	Müller-Graff et al. 2022 [41]	Deutschland	Retrospektiv	Visualisierung von prä- und postoperativen sekundären Rekonstruktionen von Flat-Panel-Volumen-CT, einschließlich der Schätzung der CDL und der Position der Elektrodenkontakte	30	Normale Anatomie	64 Jahre
32925847	George-Jones et al. 2022 [22]	USA	Retrospektiv	Vergleich der Größe der Cochlea mittels CT und MRT	21	Normale Anatomie	Keine Angabe
36294805	Li et al. 2022 [33]	China	Retrospektiv	Messung der Cochleaparameter (A-, B‑ und H‑Werte)	247	Normale Anatomie und EVAS	<18 Jahre
35970933	Weber et al. 2022 [60]	Deutschland	Retrospektiv	Vergleich von CT und MRT zur Gegenkontrolle der A‑Wert-Messung	20	Normale Anatomie	21–71 Jahre
35386404	Topsakal et al. 2022 [58]	Belgien	Klinische Studie	Bewertung der intraoperativen Genauigkeit des robotergestützten Mittelohr- und Innenohrzugangs im Hinblick auf die Entfernung zu kritischen anatomischen Strukturen (wie ChT und FN) und das beabsichtigte Ziel	22	21 normale Anatomie und 1 „incomplete partition type III“	28–83 Jahre
35193850	Ricci et al. 2022 [49]	Italien	Fallstudie	Analyse von CT-Scans mit fortgeschrittener Otosklerose und Messung der Cochleaparameter (A-, B‑ und H‑Werte)	1	Fortgeschrittene Otosklerose	73 Jahre
35032205	Di Maro et al. 2022 [17]	Italien	Retrospektiv	Umstellung von der Standardfrequenzkarte auf eine patientenspezifische Frequenzkarte	10	Normale Anatomie	14,3–78,7 Jahre
34538852	Dutrieux et al. 2022 [18]	Frankreich	Retrospektiv	Bewertung von CDL, Insertionswinkel und Insertionstiefe	106	Normale Anatomie	61 Jahre
34131770	Mertens et al. 2022 [37]	Belgien	Retrospektiv	Messung der Cochleagröße und Anwendung einer patientenspezifischen Frequenzkarte	39	Normale Anatomie	17–81 Jahre
36436080	Thimsen et al. 2022 [57]	Deutschland	Retrospektiv	Auswertung der CDL und Insertionstiefe	19	Normale Anatomie	18–75 Jahre
36514425	Bhavana et al. 2022 [6]	Indien	Retrospektiv	Auswertung der CDL und Insertionstiefe	26	Normale Anatomie	2–15 Jahre
36836405	Alahmadi et al. 2023 [2]	Saudi-Arabien	Retrospektiv	Messung von Cochleaparametern	21	EVAS	13,81 Jahre
36617441	Müller-Graff et al. 2023 [42]	Deutschland	Retrospektiv	Bewertung der Genauigkeit der radiologischen Vorhersage der postoperativen Elektrodenposition auf der Grundlage der präoperativen Bildgebung	10	Normale Anatomie	58 Jahre
36609169	Hagr et al. 2023 [24]	Saudi-Arabien	Keine Angabe	Bestimmung der besten Elektrodentrajektorie in der CI-Chirurgie anhand des rekonstruierten 3‑D-Modells und Untersuchung der chirurgischen Entfernung des retrofazialen Zugangs als direkter Zugang zum RW	25	Normale Anatomie	6,8 ± 12 Jahre

*3‑D* dreidimensional; *CDL* cochleäre Länge („cochlear duct length“); *ChT* Chorda tympani; *CI* Cochleaimplantat; *CT* Computertomographie; *DICOM* Digital Imaging and Communications in Medicine; *EVAS* Vergrößertes-vestibuläres-Aquädukt-Syndrom („enlarged vestibular aqueduct syndrome“); *FN* Gesichtsnerv („facial nerve“); *MPR* multiplanare Rekonstruktion; *MRI* Magnetresonanztomographie; *PMID* „PubMed identifier“; *RW* rundes Fenster („round window“)

### Anwendungen von Otoplan®

In den meisten Studien wurde OTOPLAN® bei normaler Anatomie genutzt. Ricci et al. [49] und Lovato et al. wendeten die Software bei fortgeschrittener Otosklerose an, Lovato et al. [35] bei verknöchertem Zustand nach einer Meningitis. Topsakal et al. verwendeten sie bei einer unvollständigen Partition vom Typ III („incomplete partition type III“), Li et al. [33] und Alahmadi et al. [2] bei einem vergrößerten vestibulären Aquädukt („enlarged vestibular aqueduct syndrom“, EVAS) und Dhanasingh et al. [16] bei einer Vielzahl von Innenohrfehlbildungen.

Die wichtigsten Ergebnisse waren (i) die Visualisierung des Innenohrs und die Messung von Cochleaparametern sowohl in der Computertomographie (CT) als auch in der Magnetresonanztomographie (MRT; 22 Studien), (ii) die Segmentierung des Mittelohrs, der Innenohrstrukturen und des Gesichtsnervs (4 Studien), (iii) die chirurgische Planung für die beste Trajektorie der Elektrodeneinführung sowie die Roboterbohrung durch den Recessus facialis (6 Studien), (iv) die Auswertung der postoperativen Bildgebung in Bezug auf die Elektrodenposition und Einführtiefe (5 Studien), (v) die Zuordnung der Frequenzen auf der Grundlage einer patientenspezifischen Anpassung (3 Studien) und (vi) die Vermessungsfunktion des Felsenbeins (1 Studie).

#### Messung der Cochleagröße

**Abb. 2:**
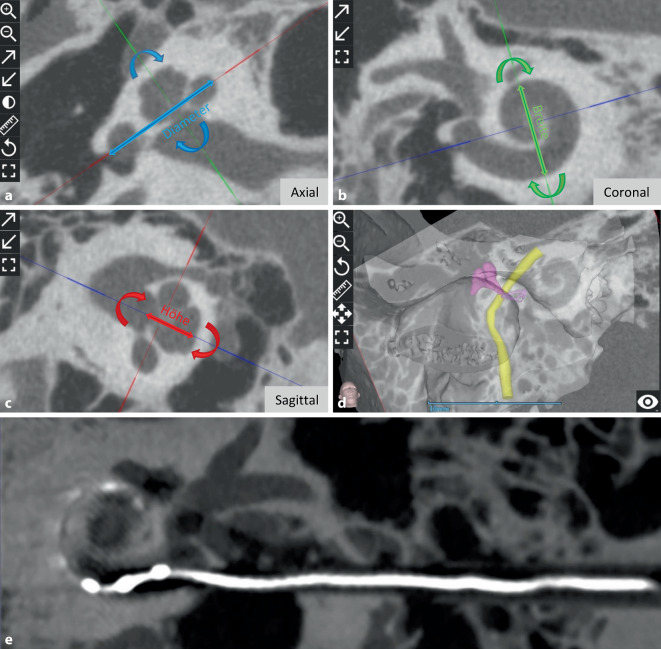
Exemplarische Darstellung der präoperativen Planung einer Cochleaimplantation und Visualisierung eines direkten Cochleazugangswegs mit inserierter Elektrode. Vermessung der Cochlea unter Verwendung der entsprechenden Parameter in der durch Rotation um die Körperachsen erzeugten „Cochleaansicht“. Axiale Ansicht (**a**), koronale Ansicht (**b**), sagittale Ansicht (**c**), dreidimensionale Darstellung des Schläfenbeins, der Gehörknöchelchen und des N. facialis (**d**). *Blauer Doppelpfeil* Durchmesser, *grüner Doppelpfeil* Breite und *roter Doppelpfeil* Höhe. Postoperatives Bild eines direkten Cochleazugangs (DCA) mit inserierter Elektrode (**e**)

Insgesamt 22 von 32 Studien haben speziell über die Bemessung der Cochleagröße an verschiedenen geografischen Standorten berichtet. Durch Drehen der 3 Körperebenen bei der Bildgebung bietet die koronale Schrägansicht („cochlear view“) eine einheitliche Standardansicht der Cochlea, mit der sich die cochleären Parameter zuverlässig messen lassen. Dabei handelt es sich um den Durchmesser (A-Wert), die Breite (B-Wert) und die Höhe (H-Wert), wie in Abb. 2a–c dargestellt, aus denen dann die CDL berechnet werden kann. Die Tab. 2 zeigt die verwendeten Bildmodalitäten und fasst die Messung der Cochleagröße anhand des A‑Werts und der CDL in mm zusammen. Diejenigen Studien, die lediglich die CDL ohne A‑Wert angegeben hatten, sollten den A‑Wert jedoch ebenfalls gemessen haben, da die CDL anhand des A‑Werts geschätzt wird.

**Tab. 2:** Datenerhebung zu Bildtypen und Cochleamessungen aus allen 32 identifizierten Studien

Studie Nr.	Autor	Prä-/postoperative Bildgebung	Bildtyp	Bildgebungsmodalität	A‑Wert (mm)	CDL (mm)
1	Lu et al. 2015 [47]	Präoperativ	CT	CBCT	–	–
2	Lovato et al. 2019 [34]	Präoperativ	CT	TBCT	–	–
3	Lovato et al. 2020 [35]	Präoperativ	CT	HRCT	–	32,4
4	Topsakal et al. 2020 [59]	Präoperativ	CT	HRCT	8,44 ± 0,4 (7,6–9,3)	–
5	Khurayzi et al. 2020 [28]	Präoperativ	CT	HRCT	7,6–10,2	–
6	Almuhawas et al. 2020 [3]	Präoperativ	CT	N/A	9,1 ± 0,27	32,5 ± 1,2
7	Jablonski et al. 2021 [27]	Prä- und postoperativ	CT	CBCT	–	33,44 (29,30–38,25)
8	Mlynski et al. 2021 [39]	Präoperativ	CT	HRCT	–	35,00 (SD ± 2,2)
9	Cooperman et al. 2021 [13]	Präoperativ	CT	N/A	–	36,2 ± 1,8
10	Spiegel et al. 2021 [55]	Präoperativ	CT	N/A	9,33 ± 0,37	34,37 ± 1,5
11	Chen et al. 2021 [11]	Präoperativ	CT	N/A	–	32,84 ± 2,0 (29,0–38,1)
12	Cooperman et al. 2021 [12]	Präoperativ	CT	CBCT	–	–
13	Niu et al. 2021 [43]	Präoperativ	CT	HRCT	–	–
14	Andersen et al. 2021 [4]	Präoperativ	CT	N/A	–	–
15	Lee et al. 2021 [31]	Präoperativ	CT	HRCT	–	32,40 ± 1,26 34,94 ± 1,20 35,77 ± 1,15
16	Auinger et al. 2021 [5]	Präoperativ	CT	HRCT und CBCT	9,3	35,82 ± 1,56
17	Kurz et al. 2022 [29]	Prä- und postoperativ	CT	fpVCT_SECO_	–	–
18	Dhanasingh et al. 2022 [16]	Präoperativ	CT	MSCT	9,0 (8,1–10,1)	–
19	Müller-Graff et al. 2022 [41]	Prä- und postoperativ	CT	MSCT, fpVCT, fpVCT_SECO_	–	34,5 ± 1,6 (31,2–36,9) 34,6 ± 1,47 (31,5–37,6) 35,84 ± 1,39 (32,9–38,4)
20	George-Jones et al. 2022 [22]	Präoperativ	CT and MRI	TBCT und MRI	–	32,7 ± 2,0 (29,4–37,6)
21	Li et al. 2022 [33]	Präoperativ	CT	HRCT	8,8 (7,4–9,7)	–
22	Weber et al. 2022 [60]	Präoperativ	CT and MRI	fpVCT und MRI	9,31 ± 0,44	36,5 ± 1,59
23	Topsakal et al. 2022 [58]	Prä- und postoperativ	CT	CBCT	–	–
24	Ricci et al. 2022 [49]	Präoperativ	CT	TBCT	–	–
25	Di Maro et al. 2022 [17]	Postoperativ	CT	HRCT	–	41,37 ± 3,1
26	Dutrieux et al. 2022 [18]	Postoperativ	CT	MSCT und CBCT	–	34,5 ± 3,5
27	Mertens et al. 2022 [37]	Prä- und postoperativ	CT	N/A	–	32,96 ± 0,73 (31,0–34,40)
28	Thimsen et al. 2022 [57]	Prä- und postoperativ	CT	MSCT und fpVCT	–	–
29	Bhavana et al. 2022 [6]	Prä- und postoperativ	CT	N/A	–	38,12 (34,2–43)
30	Alahmadi et al. 2023 [2]	Präoperativ	CT	MSCT	8,36 ± 0,32 (female) 8,82 ± 0,42 (male)	–
31	Müller-Graff et al. 2023 [42]	Prä- und postoperativ	CT	MSCT, fpVCT, fpVCT_SECO_	–	33,2 ± 2,2 33,9 ± 2,0 34,9 ± 1,8
32	Hagr et al. 2023 [24]	Präoperativ	CT	HRCT	–	–

*CBCT* Cone-Beam-Computertomographie; *CDL* cochleäre Länge („cochlear duct length“); *CT* Computertomographie; *fpVCT* Flat-Panel-Volume-Computertomographie; *fpVCT*_*SECO*_ sekundäre Rekonstruktionen des Flat-Panel Volume CT; *HRCT* hochauflösende Computertomographie („high-resolution CT“); *MRI* Magnetresonanztomographie; *MSCT* Multislice-Computertomographie; *N/A* keine Angabe; *TBCT* „temporal bone CT“

Die Messung der Cochleagröße variiert je nach radiologischer Bildmodalität und Schichtdicke

Die kleinste und größte cochleäre Größe, gemessen am A‑Wert, wurde in Tab. 2 mit 7,4 mm bzw. 10,2 mm angegeben. Die kürzeste und die längste CDL, wie in Tab. 2 angegeben, betrugen 29 mm bzw. 41,4 mm. Es ist zu beachten, dass die Messung der Cochleagröße je nach radiologischer Bildmodalität und Schichtdicke variiert [41].

Bei der Bemessung der Cochleagröße haben sich einige Studien auch mit der Intra- und Interratervariabilität der Software beschäftigt, die rundum als niedrig erachtet wird [11, 38, 41, 48]. Insbesondere ist die Studie von Chen et al. hervorzuheben, in der bei einer Messung der Cochleagröße mit OTOPLAN® eine bessere interne Konsistenz und Zuverlässigkeit im Vergleich zu einem normalen DICOM-Viewer (Digital Imaging and Communications in Medicine) nachgewiesen wurde [11]. Des Weiteren wurde in dieser Publikation als eine von wenigen eine klare Zeitangabe für die Zeit, die zum Auswerten erforderlich war, angegeben (5,9 ± 0,7 min mit OTOPLAN® im Vergleich zu 9,3 ± 0,7 min mit einem anderen DICOM-Viewer).

#### Segmentierung von Felsenbeinstrukturen

Mittel- und Innenohrstrukturen einschließlich des N. facialis können mit der Planungssoftware in wenigen Schritten segmentiert und dreidimensional dargestellt werden. Diesbezüglich sind mit den Suchkriterien der Autoren 4 Studien mit OTOPLAN® bekannt. Lu et al. berichteten 2015 über die 3‑D-Segmentierung des Gesichtsnervs mit OTOPLAN® [47]. Im Vergleich zur manuellen Segmentierung von Strukturen mit herkömmlichen Softwareprogrammen scheint OTOPLAN® Volumenunterschiede aufzuweisen. Diesbezüglich berichteten Andersen et al. über die Segmentierung der Mittelohrknöchelchen mit OTOPLAN® und verglichen die Ergebnisse mit manueller Segmentierung und automatisierten atlasbasierten Segmentierungsmethoden [4]. Topsakal et al. [59] und Hajr et al. [24] verwendeten OTOPLAN® zur Erstellung eines 3‑D-Modells der Mittel- und Innenohrstrukturen einschließlich des Gesichtsnervs und der Chorda tympani. Dies sind die Berichte über die Anwendung der OTOPLAN®-Versionen 1–3. Zum Zeitpunkt der Erstellung dieses Berichts war die Version 4.0 verfügbar, aber es wurde noch kein Bericht über die Anwendung der Version 4.0 und ihre Genauigkeit bei der 3‑D-Segmentierung anatomischer Strukturen veröffentlicht. Ein Beispiel für eine dreidimensionale Darstellung der Mittelohrstrukturen und des Gesichtsnervs ist in Abb. 2d dargestellt.

#### Trajektorie der Elektrodeneinführung sowie Roboterbohrung durch den Recessus facialis

Lovato et al. verwendeten OTOPLAN® in einer verknöcherten Cochlea, um im sog. „cochlear view“ zu visualisieren, ob der Eingang des RW verknöchert ist oder nicht [34]. Durch Auf- und Abwärtsbewegen der Schichten in der Cochleaansicht und gleichzeitiger Überprüfung der axialen Ansicht kann das Vorhandensein von Verknöcherungen in verschiedenen Ebenen der Cochlea überprüft werden. Anhand der segmentierten 3‑D-Modelle der anatomischen Strukturen kann die ideale Trajektorie für die Elektrodeneinführung geplant werden, die durch den Recessus facialis verläuft und gleichzeitig einen sicheren Abstand zum Gesichtsnerv einhält. Topsakal et al. [58] aus Belgien, Jablonski et al. [27] aus Norwegen und Auinger et al. [5] aus Österreich berichteten über den Einsatz von OTOPLAN® zur Planung eines sicheren direkten Cochleazugangs („direct cochlear access“, DCA). Das robotergestützte Bohren des DCA ist machbar, wenn man dem über OTOPLAN® ausgearbeiteten Zugangspfad folgt. Eine Schichtdicke von < 0,3 mm ist hierbei für eine sichere Trajektorienplanung erforderlich. In Abb. 2e findet sich eine beispielhafte Darstellung eines DCA-Pfads zwischen dem N. facialis und der Chorda tympani mit inserierter Elektrode.

#### Elektrodenposition

Die Software kann nicht nur für die präoperative Planung der Cochleaimplantation, sondern auch für die postoperative Lokalisationskontrolle verwendet werden. In diesem Zusammenhang berichteten bereits 5 Studien über die postoperative Elektrodenpositionierung, von diesen 5 stammen 2 aus dem Zentrum der Autoren [41, 42].

Auch für die postoperative Lokalisationskontrolle kann die Software verwendet werden

**Abb. 3:**
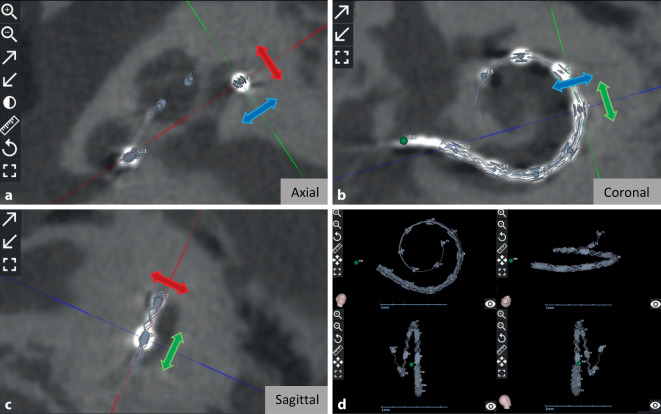
Visualisierung einer postoperativen Lagekontrolle und Bestimmung der einzelnen Elektrodenkontakte. Auswertungen in den 3 Körperebenen, axial (**a**), koronal (**b**), sagittal (**c**), und die dreidimensionale Darstellung des Implantats (**d**). Durch Verschieben der Linien in den 3 Körperebenen Kontrolle jeder Elektrode individuell in der Zentrumposition möglich (*blauer Pfeil* Durchmesserlinie, *grüner Pfeil* Breitenlinie, *roter Pfeil* Höhenlinie)

Dutrieux et al. [18] aus Frankreich berichteten über eine Winkeleinführtiefe („angular insertion depth“, AID) von 545° mit einer FLEX28-Elektrode (Fa. MED-EL). In einer kleinen Cochlea wurde mit derselben Elektrode eine AID von 565° erreicht, die in einer großen Cochlea nur 518° betrug. Bhavana et al. [6] aus Indien berichteten über eine durchschnittliche AID von 667° (Bereich: 580–773°) mit einer STANDARD-Elektrode (Fa. MED-EL). Thimsen et al. [57] aus Deutschland berichteten über eine durchschnittliche AID von 663° (Bereich: 381–798°) mit einer STANDARD-Elektrode und 581° (Bereich: 430–784°) mit einer FLEX28-Elektrode (Fa. MED-EL). Müller-Graff et al. aus Deutschland fanden heraus, dass die AID-Differenz zwischen einer präoperativen Elektrodenvorhersage und der tatsächlichen postoperativen Position abnimmt, d. h. präziser wird, wenn eine höher auflösende Bildgebung in OTOPLAN® verwendet wird, wie z. B. die sekundären Rekonstruktionen einer Flat-Panel-Volume-CT (fpVCT_SECO_) mit einer Schichtdicke von 99 µm [42]. Die Abb. 3 zeigt die postoperative Positionskontrolle der einzelnen Elektrodenkontakte innerhalb der Cochlea in den 3 verschiedenen Körperebenen (**a–c**) und in der 3‑D-Darstellung (**d**).

#### Patientenspezifische Frequenzbandzuordnung

**Abb. 4:**
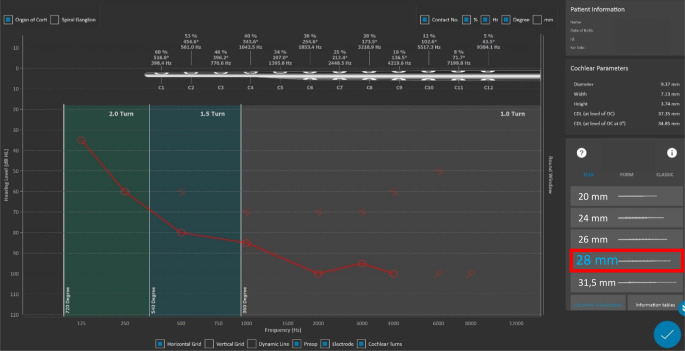
Beispielhafte Darstellung einer patientenangepassten Elektrodeninsertion in Bezug auf die elektroakustische Stimulation

Um die postoperativen Daten weiter zu nutzen, ermöglicht die Software auch die Erstellung von patientenspezifischen Frequenzbandzuordnungen. DiMaro et al. [17] aus Italien, Mertens et al. [37] aus Belgien und Kurz et al. [29] aus Deutschland berichteten über die Verwendung patientenspezifischer („cochlear size specific“) Frequenzbandzuordnungen zur Minimierung von Elektroden-Frequenz-Fehlanpassungen. Postoperative CT-Scans wurden mit OTOPLAN® ausgewertet, um die Einschubtiefe des Arrays und damit die Stimulationsposition jeder Elektrode in der Cochlea zu bestimmen. Aus der Anpassung einer patientenspezifischen Frequenzbandzuordnung ging hervor, dass die Anwendung der Mittenfrequenz auf jede stimulierende Elektrode in Kombination mit einer längeren Elektrode die Sprachunterscheidung im Vergleich zur standardmäßigen Frequenzbandzuordnung verbessert. Die Abb. 4 simuliert eine postoperative Lagekontrolle auf Grundlage der Cochleagröße und zeigt eine spezifische Frequenzzuordnung zu jeder individuellen Elektrode. Die hier generierten Frequenzkarten können über eine weitere Software (MAESTRO-Software, Fa. MED-EL) genutzt werden, um zu überprüfen, ob die einzelnen Elektrodenkontakte in den Frequenzbändern des verwendeten Audioprozessors liegen.

#### Vermessungsfunktion des Felsenbeins

**Abb. 5:**
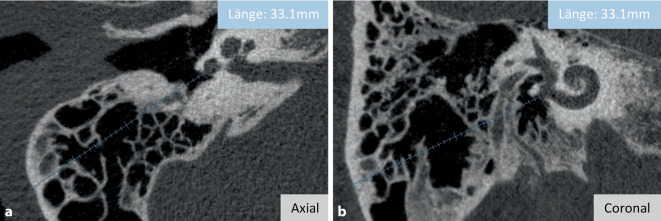
Messung der Mastoiddicke in der axialen (**a**) und koronalen (**b**) Ebene

Die angewendete Literatursuche ergab fast ausschließlich Forschungsfragen zu Anwendungen der Software, die sich mit der Cochlea beschäftigten. Nichtsdestotrotz bietet OTOPLAN® auch eine Messfunktion für alle anderen Strukturen des Felsenbeins an. In diesem Zusammenhang ergab sich durch die Literatursuche auch eine Studie, in der OTOPLAN® zur Messung der Mastoiddicke und der Schädelbreite bei CI-Patienten unterschiedlichen Alters anwendet wurde [3]. Darin wurde von einem exponentiellen Wachstum beider Messungen bis zum Alter der Pubertät berichtet, welches anschließend nahezu ein Plateau erreichte. Die Visualisierung der Messfunktion von OTOPLAN® ist in Abb. 5 beispielhaft anhand der Vermessung der Mastoiddicke sowohl in der axialen als auch in der koronalen Ebene dargestellt.

## Diskussion

OTOPLAN® ist eine otologische Planungssoftware/ein DICOM-Viewer zur Visualisierung der Felsenbeinstrukturen, insbesondere des Innenohrs und der umgebenden Strukturen. Das benutzerfreundliche Design vereinfacht den gesamten Prozess des Ausrichtens von Bildern zur Visualisierung der wesentlichen anatomischen Strukturen. Insgesamt wurden 32 Studien zum Einsatz von OTOPLAN® identifiziert, die zwischen 2015 und 2023 veröffentlicht wurden. Dabei existieren Publikationen mit oder über die Software aus vielen verschiedenen Ländern und verschiedenen Kontinenten wie Europa, Amerika oder Asien.

Die Vielzahl und weltweite Verteilung der Arbeiten deutet auf ein globales Forschungsinteresse hin und spiegelt die klinische Wertigkeit dieses Tools unter Klinikern im CI-Bereich wider. Ein Übergewicht der Studien aus dem deutschsprachigem Raum (Deutschland, Österreich und die Schweiz mit 10 von 32) ist sicherlich dem Herkunftsland der Software (Österreich) geschuldet. Dennoch scheint auch an Standorten außerhalb von Europa, wie beispielsweise Saudi-Arabien oder USA (8 von 32) zunehmend das Interesse an der Software zu steigen. Dies ist mutmaßlich auf die zunehmende Vereinfachung der Bedienung, die Zunahme von nützlichen Funktionen der Software und auf den zunehmenden Support durch die Herstellerfirma zurückzuführen, gerade im Hinblick auf das Fortschreiten einer personalisierten Medizin.

### Beurteilung der Größe der Cochlea

Von den verschiedenen, in diesem Review beschriebenen Anwendungen von OTOPLAN® wurde in 22 von 32 Studien diejenige verwendet, welche die cochleäre Größe beurteilt. Diesbezüglich bestimmt die Genauigkeit der schrägen koronalen Ebene, in der die Basalwindung der Cochlea erfasst wird, die Genauigkeit der Messung der Cochleagröße. Diese wird anhand einer Geraden bemessen, die entlang des Durchmessers der Basalwindung von der Mitte des RW zur gegenüberliegenden Seitenwand durch den zentralen Modiolus verläuft. Dieser Durchmesser der Basalwindung wird wie bereits beschrieben, bei der Cochleaimplantation auch als A‑Wert bezeichnet. Aus diesem und mitunter weiteren Parametern (B- und H‑Wert) lässt sich dann die CDL berechnen (Tab. 2). Da bei jeder Modalität (MSCT, fpVCT, fpVCT_SECO_, CBCT, HRCT, TBCT) unterschiedliche Schichtdicken verwendet werden, ist es nicht überraschend, dass die berichteten Werte etwas voneinander abweichen. Hinzu kommt, dass Populationen unterschiedlicher Regionen auf der Erde untersucht wurden und somit naturgemäß Unterschiede in der Kopfanatomie bestehen. Dabei kann die Messung nicht nur mithilfe einer CT entlang der knöchernen Wände der Cochlea erfolgen, sondern auch mit einer MRT, bei der die Parameter entlang des Flüssigkeitssignals der Cochlea gemessen werden. Dies scheint zu vergleichbaren Ergebnissen zu führen [22, 60]. Die cochleäre Größe kann also nicht nur auf Bildaufnahmen von radiologischen Geräten gemessen werden, die strahlenbasiert sind, sondern auch von solchen, die nicht strahlenbasiert sind. Dies wiederum bietet enorme Möglichkeiten, gerade bei der Implantation von Kindern, wo im besten Fall gänzlich auf Strahlung verzichtet werden sollte, da nachgewiesenermaßen eine frühkindliche Strahleneinwirkung zu einer erhöhten Rate an Komplikationen und Langzeitfolgen, wie Hirntumoren oder Katarakte, führen kann [44, 46].

Die klinische Relevanz der Ausmessung der Cochleagröße scheint enorm zu sein

Die klinische Relevanz der Ausmessung der Cochleagröße scheint enorm zu sein. War es noch vor wenigen Jahren gang und gäbe, eine standardisierte identische Elektrodenlänge für alle Cochleae zu verwenden, ist es mit der Software nun möglich, je nach Anatomie angepasste, d. h. ggf. kürzere oder längere Elektroden auszuwählen und zu implantieren. Dies führt zu einer deutlich besseren Abbildung der tonotopischen Anordnung der Sinneszellen in der Cochlea und auf lange Sicht zu besseren Hörergebnissen [50]. Des Weiteren können durch eine geeignete Elektrodenauswahl Insertionstraumata, beispielsweise durch eine zu tiefe Insertion, vermieden und ein vorhandenes Resthörvermögen, z. B. mit kürzeren Elektroden, erhalten werden. Die vielen reliablen Ergebnisse scheinen mittlerweile auch dazu zu führen, dass die präoperativen Ausmessungen der cochleären Parameter mit OTOPLAN® als Referenz dienen, um andere Forschungsfragen zu beantworten, die sich gar nicht primär mit der Software beschäftigen. Beispielsweise verwendeten Mlynski et al. die präoperativen OTOPLAN®-Daten der Cochleagröße, um zu zeigen, dass auch „electrically evoked compound action potentials“ (ECAP) zur Identifizierung der postoperativen Elektrodenposition geeignet sind [39].

### Einstellung der optimalen Messebene

**Abb. 6:**
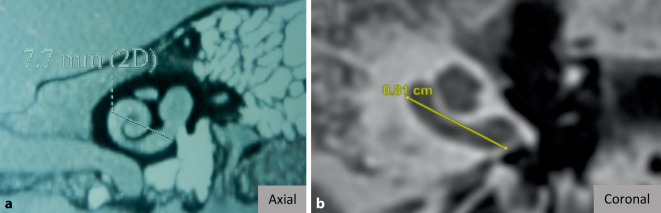
Suboptimale Ansicht der Cochlea zur Messung der Cochleagröße. Schräge koronale Ebene (**a**). (Aus [23]. Mit freundl. Genehmigung © Springer Nature, alle Rechte vorbehalten) Axiale Ebene (**b**). (Aus [38] © G. Mertens et al.; CC BY 4.0; https://creativecommons.org/licenses/by/4.0/)

Aus der Literatur geht hervor, dass die Messung der Cochleagröße in einer suboptimalen Ebene, wie in Abb. 6 dargestellt, nur dazu führt, dass falsche Maße angegeben, suboptimale Elektrodenlängen gewählt, falsche Frequenzbandzuordnungen erstellt und die Anpassungen des Audioprozessors unwirksam werden [23, 38]. Einer der Vorteile von OTOPLAN® ist die Möglichkeit, die schräge koronale Ebene in wenigen Schritten verlässlich zu erstellen. Hier zeigen sich nachgewiesenermaßen geringe Intra- und Interratervariabilitäten bei der Ausrichtung der cochleären Parameter [11, 38, 41, 48]. Als Ausblick sei darauf hingewiesen, dass darüber hinaus die neueste Version 4.0 die Möglichkeit bietet, die Größe der Cochlea automatisch zu messen, indem die Cochlea in der genannten schrägen koronalen Ebene ausgerichtet wird. Dies könnte eine noch zuverlässigere und reproduzierbare Beurteilung der Cochleagröße gewährleisten, auch wenn bislang noch keine Studien mit OTOPLAN® Version 4.0. diesbezüglich vorliegen.

### Zuverlässigkeit

Chen et al. berichteten, dass die Messung der Cochleagröße mit OTOPLAN® eine bessere interne Konsistenz und Zuverlässigkeit aufwies als mit einem normalen DICOM-Viewer [11]. Die für die Analyse jedes Ohrs mit OTOPLAN® benötigte Zeit betrug 5,9 ± 0,7 min im Vergleich zu 9,3 ± 0,7 min mit einem anderen DICOM-Viewer. Dies beweist die Effizienz von OTOPLAN® bei der Messung der Cochleagröße. Nach den Erfahrungen der Autoren, die die Software routinemäßig verwenden, ist die Zeit für die eigentlichen Messungen sogar noch kürzer und liegt eher im Bereich von 3–4 min. Es ist zu erwarten, dass bei häufigerem Gebrauch auch die Lernkurve steil ansteigt und damit der Zeitaufwand für einen geübten Anwender schnell sinkt.

### Abbildung der Frequenzverteilung

Die Messung der Größe der Cochlea ermöglicht die Abbildung der Frequenzverteilung einer individuellen Cochlea auf der Grundlage der Greenwood-Frequenzfunktion. Die postoperative CT-Bildgebung gibt Aufschluss über die während der CI-Operation erreichte Elektrodeneinführtiefe. Die Kombination dieser beiden Informationen ist nützlich für die Anpassung des Audioprozessors, indem den einzelnen Stimulationskanälen auf der Grundlage ihrer tatsächlichen Position in der Cochlea Mittenfrequenzen zugewiesen werden.

Bisher wurden Audioprozessoren anhand einer Standardfrequenzzuordnung angepasst

Bisher wurden Audioprozessoren anhand einer Standardfrequenzzuordnung angepasst [30]. Das Zentrum der Autoren hat die Hörvorteile untersucht, die mit einer anatomiebasierten Anpassung des Audioprozessors auf der Grundlage der Cochleagröße des Patienten verbunden sind. In einer Pilotstudie wurde dies bei 3 Probanden mit guter Akzeptanz durch die Probanden getestet [29]. Dies deutet auf ein großes Potenzial hin, mithilfe der OTOPLAN®-Software ein anatomiebasiertes Fitting durchzuführen und dadurch die Hörergebnisse zu optimieren. Insbesondere bei CI-Nutzern mit Unzufriedenheit hinsichtlich der Hörergebnisse oder in anderen herausfordernden Fällen könnte durch eine Neuanpassung, auch viele Jahre nach der Implantation, eine Verbesserung des CI-Hörens erreicht werden und somit die Akzeptanz eines CI weiter gesteigert werden.

### Planung der Bohrtrajektorie

Der Einzug der Robotik in den CI-Bereich ist sowohl für die CI-Chirurgie als auch für die Anpassung von Audioprozessoren zunehmend von Interesse. Damit der Roboter sicher durch den Recessus facialis bohren kann, um die Cochlea zu erreichen, ist OTOPLAN® hilfreich bei der Planung der Bohrtrajektorie, ohne den Gesichtsnerv oder die Chorda tympani zu verletzen. Dieses Verfahren wurde von CI-Chirurgen bei mehr als 20 Patienten erfolgreich eingesetzt, wobei kein Fall einer Verletzung des Gesichtsnervs gemeldet wurde, was die Wirksamkeit von OTOPLAN® bei der Vermessung anatomischer Strukturen belegt [58]. Die manuelle Segmentierung der anatomischen Strukturen erfordert Geduld und Wissen, um die wichtigen Strukturen sorgfältig zu erfassen und die 3‑D-Bilder zu erstellen. Die automatische 3‑D-Segmentierung des Innenohrs und der umgebenden Strukturen durch OTOPLAN® ist sehr praktisch, insbesondere für junge, wenig erfahrene Kliniker, um die Anatomie und Orientierung der Strukturen zu verstehen.

### Vermessung von Felsenbeinstrukturen

Bezugnehmend auf die Vermessungsfunktion von Felsenbeinstrukturen der OTOPLAN®-Software lässt sich festhalten, dass diese Funktion bislang erst in überschaubarem Maße wissenschaftlich genutzt wurde. Eine Studie wurde zur Messung der Mastoiddicke und der Schädelbreite bei CI-Patienten unterschiedlichen Alters verwendet [3]. Hier wurde über ein exponentielles Wachstum beider Messungen bis zur Pubertät berichtet. Ähnliche Ergebnisse zeigen sich bei Chen et al., die ohne Hilfe einer Software die Mastoiddicke vermessen haben [10]. Dies deutet auf eine zuverlässige Vermessungsfunktion von OTOPLAN® hin. Insgesamt scheint diese Funktion durchaus Potenzial zu haben, den Kliniker sinnvoll zu unterstützen, beispielweise bei der Vermessung der Mastoiddicke hinsichtlich der Planung bei der Implantation von Knochenleitungsimplantaten.

#### CI-spezifischer DICOM-Viewer

CT-Aufnahmen des Felsenbeins sind seit 1980 verfügbar, und es gab mehrere Forschungsarbeiten, in denen die anatomischen Variationen des Innenohrs und der umgebenden Strukturen mit Standard-DICOM-Viewern untersucht wurden [53]. Im Laufe der Zeit entwickelten sich immer mehr Ansätze, um die Längenmessung der Cochlea auf radiologischen Bildern durchzuführen, insbesondere mathematischer Art und in Form von 3‑D-Projektionen [19, 26, 52]. Auch entstanden Forschungs-Softwares wie beispielsweise die kostenlosen medizinischen Image-Viewer „Horos“ oder „3D Slicer“ (Open-Source-DICOM-Viewer). Diese wurden insbesondere bei der cochleären Längenmessung mittels der multiplanaren Rekonstruktion verwendet, die vergleichbare Ergebnisse wie bei der Ausmessung mit OTOPLAN® ergaben [51]. Es bestand jedoch ein Bedarf an einem CI-spezifischen DICOM-Viewer mit Funktionen, die dem Kliniker die Arbeit erleichtern. OTOPLAN® ist die erste Software ihrer Art mit CE-Kennzeichnung, die in der klinischen Praxis eingesetzt wird. Eine andere kürzlich vorgestellte CI-spezifische Software ist die Software Oticon Medical Nautilus (Fa. Oticon A/S, Smørum, Dänemark) die ebenfalls eine automatisierte Bildverarbeitung verwendet [36]. Diese ist allerdings nicht CE-zertifiziert und aktuell nur als Forschungsplattform für Studien im Zusammenhang mit einer CI-Versorgung verfügbar. Somit verbleibt derzeit als klinisch anwendbar nur die OTOPLAN®-Software, die sich im Laufe der Zeit mit einer guten Akzeptanz im CI-Bereich weiterentwickelt hat und den Studien aus diesem Review zufolge weltweit Anerkennung gefunden hat.

## Fazit für die Praxis

- Diese umfassende Literaturübersicht umfasst 32 Studien, die über die verschiedenen Anwendungen der Software OTOPLAN® im Rahmen einer Cochleaimplantation berichteten und zwischen 2015 und 2023 veröffentlicht wurden.
- Diese Software wird häufig für die genaue Beurteilung der Größe der Cochlea verwendet, die bekanntermaßen in der menschlichen Bevölkerung variiert. Hierzu sollte klinischerseits die höchstmögliche Bildauflösung, wie bspw. „sekundäre Rekonstruktionen des flat-panel volume CT“ (fpVCT_SECO_) mit 99 µm, angestrebt werden, da sie die genauesten Messungen mit geringer Intra- und Interratervariabilität ermöglicht.
- Zudem wird OTOPLAN® auch für die postoperative Beurteilung der Elektrodeneinführtiefe und die Anwendung einer patientenspezifischen Frequenzbandzuordnung bei der Anpassung von Audioprozessoren eingesetzt. Dies könnte insbesondere in Hinblick auf ein anatomiebasiertes CI-Fitting von erheblicher Relevanz sein und in Zukunft zu einem noch weiter verbesserten Höreindruck führen.
- OTOPLAN® ist bisher der einzige CE-gekennzeichnete DICOM-Viewer (Digital Imaging and Communications in Medicine) für den CI-Bereich, der prä-, intra- und postoperative Bilder verarbeiten kann.
- Dies wird den klinischen Arbeitsablauf einer erfolgreichen Cochleaimplantation auch in Zukunft enorm unterstützen.
